# What do we measure when we measure cell-associated HIV RNA

**DOI:** 10.1186/s12977-018-0397-2

**Published:** 2018-01-29

**Authors:** Alexander O. Pasternak, Ben Berkhout

**Affiliations:** Laboratory of Experimental Virology, Department of Medical Microbiology, Academic Medical Center of the University of Amsterdam, Meibergdreef 15, 1105 AZ Amsterdam, The Netherlands

**Keywords:** Cell-associated HIV RNA, Virological biomarker, HIV persistence, HIV reservoir, Antiretroviral therapy, HIV cure

## Abstract

Cell-associated (CA) HIV RNA has received much attention in recent years as a surrogate measure of the efficiency of HIV latency reversion and because it may provide an estimate of the viral reservoir size. This review provides an update on some recent insights in the biology and clinical utility of this biomarker. We discuss a number of important considerations to be taken into account when interpreting CA HIV RNA measurements, as well as different methods to measure this biomarker.

## Introduction

To express all its genes, HIV produces a large number of differentially spliced transcripts collectively termed “cell-associated (CA) HIV RNA” [1, 2]. In HIV-infected individuals, especially those on suppressive antiretroviral therapy (ART), CA HIV RNA is an important surrogate marker of the viral reservoir and the response to ART [3–5]. Recent years have seen considerable interest in quantifying CA RNA as a measure of HIV latency reversion, therefore it has been used as a readout in a number of clinical trials aimed at HIV remission [6–9]. For the correct interpretation of the outcomes of such trials, it is necessary to understand the meaning of CA RNA measurements. In 2013, the significance of CA HIV RNA as a biomarker of viral persistence was summarized in an extensive review article [10]. This review provides an update on some recent insights in the biology and clinical utility of this biomarker.

## Transcription versus production versus replication: What do we measure?

By establishing latent infection, HIV forms a long-lived reservoir in infected individuals, which persists despite suppressive ART and is currently considered the main obstacle to an HIV cure [11, 12]. Accordingly, complete eradication of the reservoir would signify a sterilizing cure, and a substantial degree of reservoir depletion would likely be necessary for achieving a state of prolonged ART-free HIV remission, otherwise termed “functional cure” [13]. Depleting the reservoir is therefore the principal goal of HIV curative strategies, of which the so-called “shock-and-kill” approach has received most attention in recent years [14, 15]. The idea behind “shock-and-kill” is to reverse HIV latency with specific compounds, termed “latency-reversing agents” (LRA). The resulting switch to productive infection, or at least the elevated level of HIV protein expression, would then subject the HIV-infected cells to immune-mediated clearance and/or viral cytopathic effects, and the free virions generated in the process would be unable to establish productive infection of new cells in the setting of completely suppressive ART. Because HIV latency has traditionally been understood as transcriptional latency (persistent replication-competent proviruses that are transcriptionally silent but can be reactivated to produce infectious virus particles and reignite viral spread in the absence of ART), most LRAs currently in clinical or preclinical use function by stimulating HIV transcription with minimal cellular activation [16]. Logically, CA HIV RNA has been used as a measure of the potency of these compounds for latency reversion, both ex vivo and in several clinical trials [6–9, 17, 18]. Modest increases in CA RNA level, sometimes accompanied by elevated cell-free HIV viremia, have indeed been observed in these trials, but despite this, no substantial reservoir reduction has been measured so far in most studies. Here it must be noted that HIV transcription does not necessarily lead to productive infection, consequently an increased CA RNA level does not automatically mean full reversion of latency. Two possible reasons for this are that (1) part of the transcripts is defective at the sequence level for production of active viral proteins and infectious particles (discussed in detail below), and (2) latency might be regulated not only at the transcriptional but also at multiple post-transcriptional levels (e.g. splicing and nuclear export of viral RNA, translation, viral particle assembly and maturation, etc.). For instance, Chun et al. could not detect cell-free virions in ex vivo resting CD4+ T cell cultures from ART-treated individuals, despite the presence of CA RNA [19]. Furthermore, Hong et al. did not find any correlation between CA RNA levels and residual plasma viremia in ART-treated subjects [20], although a weak correlation was observed by Li et al. [21]. This suggests that in the setting of suppressive ART, at least some HIV transcription events do not result in virus production. This idea is supported by the large disproportion between the decay kinetics of cell-free and CA HIV RNA upon ART initiation [2, 4, 22–25].

By sensitive methods, CA HIV RNA can be detected in the vast majority of peripheral blood mononuclear cell (PBMC) or CD4+ T-cell samples isolated from HIV-infected individuals on prolonged ART in the absence of any ex vivo stimulation [3–5, 26], and the concept of “leaky latency” has recently been put forward to explain these findings (CROI 2015). However, this concept assumes only transcriptional latency (that can occasionally “leak”), ignoring the possibility of post-transcriptional blocks to HIV expression. In general, though, virus latency does not require complete shutdown of viral gene expression, only lack of infectious progeny production [27]. In fact, already in 2011 Pace et al. [28] proposed the “continuum of latency” with barriers to productive infection at different stages of the viral replication cycle in different populations of latently infected cells, and in 2012 we introduced the concept of the “active HIV reservoir” to describe latently infected cells that are actively transcribing HIV RNA but do not produce infectious virus particles [3, 29]. A question then arises of whether the active reservoir is a true reservoir, in other words how would such cells persist for a long time and escape immune-mediated killing. One simple possibility is that no viral proteins are produced despite ongoing HIV transcription. In a primary CD4+ T-cell model, Mohammadi et al. [30] observed disproportionally low activation of viral translation compared to activation of viral transcription by the LRA vorinostat. As mentioned above, several mechanisms of post-transcriptional regulation of HIV gene expression are possible. For instance, multiply spliced HIV RNA has been shown to be retained in the nucleus both in resting CD4+ T cells from patients on ART and in a chemokine (CCL19) induced model of HIV latency in primary resting CD4+ T cells [31, 32]. This nuclear localization of multiply spliced RNA precluded high-level transcription and nuclear export of other CA HIV RNA species and protein translation and possibly contributed to the latent state of HIV in these cells. In addition, differential expression of Rev cofactors such as Matrin 3 or PSF in different cell types can contribute to blocking HIV RNA nuclear export [33–35]. Furthermore, Li et al. [36] reported selective inhibition of HIV translation by host factor Schlafen 11 in a codon usage-dependent manner, and inhibition of HIV replication by a number of host microRNAs has been observed, although the precise mechanism of their action is still unclear [37, 38]. However, several groups recently reported the detection of HIV Gag protein expression in ART-treated individuals, albeit Gag-positive cells were much less frequent than cells containing CA RNA [39–41]. Future research will reveal whether more sensitive assays are able to detect HIV proteins in a larger proportion of cells. In any case, one mechanism of persistence of such HIV protein-expressing cells could be the large prevalence of CTL escape mutations in the latent reservoir, recently demonstrated for Gag epitopes [42] but likely present in other HIV proteins as well.

As discussed above, although HIV transcription is a prerequisite for virus production, the mere presence of CA HIV RNA, or an increase in its copy number in a cell from an ART-treated individual does not automatically signify an increase in infectious virus production and this should be taken into account when designing latency reversion experiments. Even less does residual HIV transcription per se mean residual virus replication (discussed in detail in [10]). The debate about the possibility of residual HIV replication despite ART has been ongoing for a long time [43], but recently gained some renewed momentum with the publication of an intensive longitudinal study of HIV evolution in lymphoid tissue that revealed a temporal structure of viral populations during early ART [44], although other investigators challenged that conclusion [45, 46]. Furthermore, two recent reports revealed suboptimal tissue concentrations of antiretroviral drugs that negatively correlated with the slower decay or increases in the follicular dendritic cell network-associated virions and detection of viral RNA in productively infected cells [47, 48]. Combined, this evidence points to the possibility of low-level HIV replication in tissues due to suboptimal ART penetration, even if infection of new cells is completely suppressed in peripheral blood. This possibility also has to be taken into account during LRA clinical trial design. In a number of clinical trials of different LRAs (vorinostat, disulfiram, panobinostat), investigators observed a persistent “post-dosing effect” on CA HIV RNA, with increased CA RNA levels detected long after the measures of LRA pharmacodynamics have returned to baseline levels [6–8]. To explain this phenomenon, it has been argued that LRAs could exert a long-lasting effect on host gene expression that could influence viral RNA levels [6], but given the different mechanisms of action of the LRAs studied, this is unlikely to be the only explanation. An alternative explanation could be that the free virus particle production induced by the LRA treatment could lead to low-frequency de novo infections, at least in tissues and anatomic compartments where ART pressure might be suboptimal. The newly infected, activated CD4+ T cells would in turn produce virus to infect other cells, and in this manner, a limited chain of new infections could continue for some time. If this LRA-induced residual replication occurs, CA RNA, even measured in peripheral blood, is expected to be a very sensitive marker of this process. This is because (1) a productively infected cell can contain hundreds to thousands of HIV unspliced RNA copies at the peak of infection [49], (2) some cells could be infected by cell-to-cell contact without free virion release [50], and (3) infected cells can traffic between tissues and periphery [1, 51]. In the disulfiram trial, the highest LRA dose used (2000 mg) caused a significant post-dosing increase not only in CA RNA but in plasma HIV RNA level as well [7]. Interestingly, low-level plasma viremia after recombinant HIV poxvirus vaccination, another intervention that is expected to activate latent HIV, has been shown previously to correlate with HIV sequence evolution, suggesting that HIV activation might cause some residual replication [52]. Another relevant observation is that vorinostat has been shown to increase susceptibility of primary CD4+ cells to HIV infection, whereas the other LRA romidepsin has an opposite effect [53, 54]. Remarkably, the post-dosing effects described above have been observed in the clinical trials of vorinostat but not romidepsin [6, 7, 9, 55]. This putative side-effect of reservoir activation argues for a strict ART adherence during the trials, as small deviations from optimal adherence could lead to residual virus replication, even if plasma viremia stays undetectable by commercial assays [3, 56]. In the worst-case scenario, this residual replication could lead to significant replenishment of the HIV reservoir, compensating for any LRA-induced reservoir depletion, and this might be one of the reasons behind the lack of efficacy in LRA trials so far.

## Unspliced versus spliced versus poly(A): What should we measure?

More than 100 different transcripts can be derived from the HIV unspliced (US) genomic RNA by alternative splicing, although it is unclear if all of them are present in infected individuals [57, 58]. These include incompletely, or singly spliced and completely, or multiply spliced (MS) transcripts that can be roughly divided to 1, 2, and 4 kb classes [57]. The 2 kb MS RNAs encode the regulatory proteins Tat, Rev, and Nef. Of these, Tat is necessary for high-level HIV transcription, whereas Rev is needed for efficient nuclear export of US and incompletely spliced HIV RNA species that encode the structural and accessory viral proteins (reviewed in [59]). The relative abundance of HIV RNA species has been studied in different experimental systems. In H9 cells, MS RNA production has been shown to peak at the early stages of the HIV replication cycle, after which incompletely spliced and US RNA took over [60]. Similar temporal dynamics has been observed after stimulation of ACH-2 cells with PMA [61]. This can be explained by the restriction of Rev function in these cells, so that a high MS RNA (and consequently Rev) level must be achieved before US RNA can be efficiently exported to the cytoplasm, thereby escaping splicing or degradation. More recently, by deep sequencing of RNA derived from primary cells 48 h post-infection with the HIV_89.6_ strain, the Bushman group could determine that the relative abundance of MS RNA was close to that of US RNA, and Mohammadi et al. observed a similar pattern in their primary CD4+ T-cell model [30, 57, 62]. However, in resting CD4+ T cells directly infected by spinoculation, Pace et al. detected a large excess of US RNA over incompletely spliced and MS RNA species [63], suggesting a possible limitation in splicing factor availability and/or RNA nuclear export defect in these cells. This complements the reports that described the predominance of short abortive transcripts in resting CD4+ cells [64–66], and “long” but incomplete transcripts in CD4+ cells from ART-treated individuals [67], presumably due to inefficient transcription elongation.

In HIV-infected individuals, ART-treated or not, MS RNA is usually measured to be much less abundant than US RNA [4, 5, 68–70], although it is unclear to what extent this reflects underestimation of MS RNA copy numbers by qPCR due to primer/probe-template mismatches that could be more frequent in *tat/rev* than in *gag* assays. It should be noted, however, that the difference in relative abundance between US and MS RNA was observed even when patient-matched primers and probes were used for qPCR [5, 71]. In individuals on prolonged ART, US RNA is readily detectable but MS RNA is difficult to detect unless cells are stimulated ex vivo. Nevertheless, MS RNA is detectable early on ART and a higher US/MS RNA ratio at 12 weeks ART was shown to be predictive of a reduced immunological response to therapy at 48 and 96 weeks and correlated with markers of CD4+ T-cell activation and apoptosis [72]. Interestingly, in untreated patients the US/MS ratio is lower in long-term nonprogressors and has been shown to correlate with rapid progression [73–76]. In light of the temporal shift from the MS to US RNA expression discussed above, a higher US/MS RNA ratio in a patient might reflect the higher frequency of HIV-infected cells in the later stages of viral the replication cycle, which is characterized by expression of viral structural proteins and presentation of antigens. Such cells could exert pressure on the host immune system, causing persistent immune activation and apoptosis and contributing to poor immunological response to ART. Further research will show whether the US/MS RNA ratio could be used as a marker of residual HIV pathogenesis on ART.

Another issue that is relevant to the latency reversal studies is which CA HIV RNA species would be a better surrogate for measuring the LRA efficacy and changes in replication-competent reservoir. Both US and MS RNA have been used in this role in inducible HIV transcription assays (see below and the review article by Plantin et al. in this Special Issue [77]). It has been argued that MS RNA could be a better surrogate for the replication-competent reservoir as splicing requires the presence of several cis-acting sequences in the HIV genome and therefore the presence of MS RNA reduces the possibility of measuring proviruses with large deletions [78]. The relative decrease in MS RNA level upon ART initiation is more prominent than that of US RNA [79–82], and cells containing measurable MS RNA are much rarer under ART than those containing US RNA [5, 71]. This suggests that MS RNA-positive cells can indeed be a more proximal surrogate of cells containing HIV proviruses that are reactivated from latency, at least to some extent. This is confirmed by the recent data from Yukl’s group, as they observed much stronger increases in MS RNA than in “long” HIV transcripts upon ex vivo stimulation of CD4+ T cells [67]. However, even despite reactivation, many such proviruses will still be unable to establish the productive infection and release infectious progeny due to various genetic defects. This is the reason why measurements of frequencies of cells that can be induced to express any HIV RNA species will always overestimate the replication-competent reservoir size.

One more issue regarding the choice of which HIV RNA species to measure as a surrogate of latency reversion is whether the transcripts measured by the *gag* assays represent genuine viral RNA. As HIV preferentially integrates within actively transcribed host genes [83], Bullen et al. recently proposed that some transcripts detected by the *gag*-specific assays may not represent bona fide HIV RNA but rather chimeric host-HIV readthrough transcripts that are transcribed from upstream host promoters [84]. They demonstrated that vorinostat could activate such readthrough transcription to the similar levels as *gag* transcription and proposed to use the polyadenylated HIV mRNA-specific assay to detect genuine HIV RNA [84, 85]. However, neither the absolute copy numbers of readthrough and *gag* RNA, nor the readthrough/*gag* RNA ratio was presented and therefore the contribution of host transcripts to the *gag* RNA pool remained unclear. Subsequently, we and others demonstrated that in ART-suppressed individuals, this contribution is very modest and that the vast majority of HIV *gag* RNA transcripts represent genuine HIV unspliced RNA [67, 86]. However, recent data from Yukl’s group suggests that most of these transcripts might still be incomplete and therefore an advantage of measuring the poly(A) HIV mRNA would be that such incomplete transcripts are avoided [67]. A disadvantage of the latter assay is that it does not discriminate between unspliced and spliced HIV RNA and therefore is of limited use in HIV reservoir studies.

## Early versus late: When should we measure?

It has been firmly established that early initiation of ART limits the HIV reservoir size [87, 88]. HIV-infected individuals who start ART during acute or early infection achieve lower CA RNA levels than those who start therapy during chronic infection [80, 89–91]. Early ART preserves immune functions and limits the possibilities for HIV to escape from the host CTL response [42], providing a likely explanation for the smaller active reservoir under early therapy. However, all these previous studies compared different patients. We recently undertook a longitudinal study to compare viral reservoirs in the same patients treated in two phases during early and chronic HIV infection, and assessed the long-term effects of early therapy on the HIV reservoir during treatment initiated at chronic infection [92]. We quantified levels of CA US RNA and total HIV DNA in HIV-infected individuals who had participated in a randomized controlled trial of 24 or 60 weeks of temporary ART versus no treatment during primary HIV infection (Primo-SHM study; [93]) and subsequently (re)started therapy during chronic infection after an average of 2 years without treatment. As demonstrated earlier, levels of both US RNA and total DNA during early ART were significantly lower than levels of corresponding markers during ART initiated at chronic infection in patients who were not treated with early ART. Surprisingly, however, no significant difference was found between the levels of CA RNA or DNA measured during early and chronic therapy periods in the same patients, and strong correlations in the HIV RNA and DNA levels between the two therapy periods were observed. Finally, the level of US RNA, measured during chronic infection ART, was significantly lower in patients who had been pre-treated during primary infection than in patients who had not been pre-treated. Taken together, these data suggest that early ART, even when interrupted, has a long-term suppressive effect on the viral reservoir during treatment initiated subsequently during chronic infection. This observation might be reassuring for HIV-infected individuals participating in HIV curative interventions who sometimes have to temporarily interrupt ART [94].

Another intriguing observation related to the timing of HIV RNA measurement is that CA RNA levels might actually fluctuate with the time of the day. This observation was made during the phase II clinical trial of disulfiram [7]. Significantly higher CA RNA levels have been detected in one out of three baseline samples that was collected earlier in the day than the two others from the same trial participants (all three samples were collected prior to the intervention). To explain this finding, the authors hypothesized that HIV transcription could be influenced either by circadian rhythm or by anticipatory stress (unlike two others, the sample with higher CA RNA levels was collected immediately prior to disulfiram treatment). Recently, data have been presented that indeed confirm the effect of stress on HIV transcription [95]. It is important to understand whether the observed effect was HIV-specific or was reflecting the changes in host transcription in response to either stimulus. HIV RNA is transcribed by RNA polymerase II, which activity is known to fluctuate according to the circadian rhythm ([96] and references therein). Furthermore, psychosocial stress has been shown to rapidly activate NF-κB [97, 98], and core circadian protein CLOCK is a positive regulator of NF-κB-mediated transcription [99]. As NF-κB is a known HIV transcription factor [100–102], this might be the mechanism behind the effect of stress and/or circadian rhythm on CA HIV RNA. In the studies of Elliott et al. [7] and Hecht et al. [95], CA RNA levels were normalized to 18S ribosomal RNA, which is transcribed by RNA polymerase I and it is unknown whether this enzyme is regulated by circadian rhythm or stress. Therefore, to disentangle the host- and virus-specific effects, it would be informative to assess the expression of host RNA polymerase II-transcribed genes in parallel with HIV, ideally genes that are responsive to NF-κB. In summary, these findings add another dimension to the longitudinally fluctuating nature of CA HIV RNA levels under ART as shown previously [3, 103], and this has to be taken into account when interpreting the results of latency reversion trials.

## Tissue versus periphery: Where should we measure?

Apart from peripheral blood, CA HIV RNA has been measured in human gut-associated lymphatic tissue (GALT), lymph nodes, and tonsil tissue [69, 104–106], as well as in various tissues of humanized mice [107, 108]. In addition, CA RNA of SIV or SHIV has been measured in non-human primate models of HIV, by PCR-based or in situ hybridization-based methods [48, 109–112]. As more than 98% of the body CD4+ T cells are locked in the lymphoid organs, these sites are the primary sites of HIV replication in untreated HIV-infected individuals. It is unclear, however, whether the infection frequency in tissue is higher than in peripheral blood. In ART-treated individuals, Yukl et al. reported significantly higher total HIV DNA levels normalized to CD4+ cells in multiple GALT sites compared to the peripheral blood, whereas for CA RNA, a significantly higher level has been observed for the ileum only [105]. In the subsequent study by the same group, CA RNA levels were reported to be even lower in the rectum than in peripheral blood in CD4+ T cells and total white blood cells, despite higher HIV DNA levels [104]. Interestingly, in these studies, the relative HIV transcription level (RNA/DNA ratio) tended to be higher in peripheral blood than in the rectum, although levels of T-cell activation in the blood were clearly lower. One explanation for these reduced RNA/DNA ratios in the gut might be that the measured total DNA could include a significant portion of incomplete reverse transcripts and complete but unintegrated DNA, reflecting possible recent infections due to suboptimal tissue antiretroviral drug levels and/or cell-to-cell HIV transfer [47, 50, 113, 114]. The distribution of HIV DNA and RNA in T-cell subsets was also different between gut and periphery: most HIV DNA and RNA in the peripheral blood were found in CCR7+ cells, whereas in the gut, most HIV DNA and RNA were found in effector memory cells [104]. This differential HIV distribution was confirmed by an independent study [115]. The latter study did not find a statistically significant difference in infection frequency between GALT and blood. Another study by the same group could not measure a significant difference between the infection frequencies in memory CD4+ cells from lymph nodes and peripheral blood in untreated HIV-infected individuals, although there was a trend to higher infection levels in tissue [116]. However, significantly higher CA US RNA levels were observed in memory CD4+PD-1+ T cells isolated from lymph nodes compared with blood [106]. In the lymph node, cells with high expression of the cell surface receptors CXCR5 and PD-1 correspond to follicular T helper (T_FH_) cells that are a highly specialized subset of T helper cells that reside in lymph node germinal centers. Both in untreated and treated HIV-infected individuals, T_FH_ cells were shown to harbor higher levels of HIV DNA and RNA than other memory CD4+ T-cell subsets, and CA RNA levels in these cells inversely correlated with the duration of treatment [106, 117]. As germinal centers were shown to be an immunologically privileged site with restricted CTL function [118], persistent CA RNA transcription there could be a source of infectious virus production and fuel virus rebound upon ART interruption. Indeed, T_FH_ cells were demonstrated to be enriched in replication-competent proviruses in ART-treated individuals [106], although we still do not know whether the frequency of replication-competent proviruses correlates with CA RNA levels. Further research should reveal whether T_FH_ cells are enriched in “intact” proviruses compared with other T-cell subsets and whether virus evolution continues under ART in these cells in lymph nodes. Finally, it should be noted that for obvious reasons, all studies mentioned above were small and therefore the results should be interpreted with some caution.

## Bulk versus single-cell, in situ hybridization versus PCR, digital PCR versus qPCR: How should we measure?

A plethora of different methods have been developed to measure CA HIV RNA (Table 1). The simplest, cheapest, and quickest methods are based on the quantitation of CA RNA in bulk cellular extracts by reverse transcription (RT)-qPCR [5, 85, 119–121], to which a nested or seminested pre-amplification step can be added to increase the assay sensitivity [4, 122]. Over the last 10 years, seminested RT-qPCR has been extensively used to measure CA HIV RNA in different clinical cohorts and helped obtain important insights into the clinical value of this biomarker for monitoring ART response and LRA efficiency [3, 4, 6, 8, 29, 68, 72, 86, 92]. Apart from increasing sensitivity, the addition of the pre-amplification step results in higher accuracy in the lower quantitation range and better tolerance of RT or PCR inhibition than single-step qPCR-based assays, while not requiring much extra time and labor [122]. However, the addition of one or two extra primers increases the probability of underestimation of target copy numbers in samples from HIV-infected individuals due to primer/probe-template mismatches. In addition, absolute quantitation by qPCR relies on the external standards and therefore qPCR-based assays are difficult to standardize between different laboratories.

**Table 1:** Assays to measure CA HIV RNA in HIV-infected individuals

Bulk or single-cell?	Chemistry	Assay type	Pros	Cons	References
Bulk	qPCR	RT-qPCR	Sensitive, high throughput, low cost	Depends on external standards, no single-cell analysis possible, sensitive to primer/probe-template mismatches	[85, 119, 121]
		RT-seminested qPCR	Highly sensitive, high throughput, increased accuracy in the lower quantitation range, low cost	Depends on external standards, no single-cell analysis possible, sensitive to primer/probe-template mismatches	[4, 6, 122]
	Digital PCR	RT-ddPCR	Sensitive, high throughput, no standard curve needed, less affected by primer/probe-template mismatches	False positive droplets, possible underestimation due to molecular dropout, external calibrator needed to account for the yield of the RT reaction, higher cost compared to qPCR, no single-cell analysis possible	[26, 65]
Single-cell	PCR	Limiting dilution-RT-qPCR or -RT-ddPCR	Estimates frequencies of CA RNA+ cells	Costly, laborious, in its current form does not report CA RNA copy numbers per cell	[78, 130, 131]
		Limiting dilution-RT-PCR-sequence analysis	Estimates frequencies of CA RNA+ cells and CA RNA copy numbers per cell, allows to estimate intactness/defectiveness and clonal expansion of expressed proviruses	Costly, laborious, does not directly measure frequencies of CA RNA+ cells and CA RNA copy numbers per cell, only 15% of HIV genome sequenced so mismatches outside this region cannot be accounted for	[133]
		Single-cell-in-droplet-ddPCR	High-throughput, allows direct quantitation of CA RNA+ cells, lower cost compared to other single-cell methods	In its current form does not report CA RNA copy numbers per cell, possible underestimation of CA RNA+ cell numbers due to “cellular dropout”	[135]
	In situ hybridization	Microscopy-based	Allows direct visualization and characterization of CA RNA+ cells, duplex HIV RNA and DNA detection in the same tissue section possible, low background	Does not report CA RNA copy numbers per cell, possibly reduced sensitivity compared to PCR-based assays, laborious, relatively low throughput	[48, 141]
		Flow-based	Allows characterization of CA RNA+ cells, can be combined with HIV protein detection	Does not report CA RNA copy numbers per cell, reduced sensitivity compared to PCR-based assays, high background	[40, 142, 143]

With this in mind, efforts have been made to develop digital PCR-based methods to measure HIV DNA and RNA, as digital PCR is by definition an absolute DNA quantitation method that does not require a standard curve (see the review of Rutsaert et al. in this Special Issue [123]). For HIV DNA quantitation, Strain et al. [124] demonstrated the superiority of droplet digital PCR (ddPCR) over single-step qPCR in precision and accuracy, with an added benefit of better tolerance of target sequence variation compared to qPCR. However, for quantitation of CA US and MS HIV RNA, Kiselinova et al. [26] reported better quantitative linearity, accuracy and sensitivity of seminested qPCR compared to ddPCR, especially in the lower quantitation ranges. On the other hand, ddPCR in that study could detect MS RNA in a larger proportion of samples from ART-treated individuals than qPCR, although the detection rate of MS RNA in samples from untreated individuals was equal between the methods and both methods demonstrated equally high detection rate of US RNA on and off ART. The caveat, though, is that a number of studies, including Kiselinova et al., reported positive droplets in some no-template control wells in digital PCR, undistinguishable by fluorescence from the positive droplets in positive control wells [26, 124, 125]. The origin of these false-positive droplets is presently unclear but they greatly complicate the use of digital PCR for quantitation of extremely low target copy numbers, such as observed for CA HIV RNA from ART-treated individuals. Setting up a detection threshold based on the maximal number of positive droplets in the no-template control wells might alleviate this problem but will substantially compromise the assay sensitivity. For example, in this case, all samples from ART-treated individuals that were scored positive for MS RNA by ddPCR in the study by Kiselinova et al. [26] would be scored negative. qPCR does not have this issue and therefore is preferable to use when low target copy numbers are expected. Another drawback of digital PCR is possible underestimation of target copy numbers due to molecular dropout, when the target molecule is present in the partition but is not amplified [126]. Finally, it is important to realize that unlike the quantitation of DNA by digital PCR, which is absolute, the quantitation of RNA still needs an external calibrator to account for the yield of the RT reaction which can vary widely depending on the priming strategy, reaction conditions, and the enzyme used [127, 128]. Digital PCR measures cDNA, not RNA, and the absence of such calibrator might cause another significant underestimation of the target RNA copy numbers. This is likely true even for one-step RT-digital PCR methods, where the RNA sample is partitioned prior to RT [129], and this makes the digital PCR-based quantitation of RNA as dependent on the external standard as the qPCR-based methods.

As discussed above, bulk PCR-based methods of CA HIV RNA measurement are highly sensitive, high-throughput, and inexpensive, and therefore perfectly suited for the analysis of HIV transcription levels in a large number of samples. However, for in-depth dissection of HIV reservoir these assays are of limited use, because they do not allow determining frequencies of HIV RNA+ cells, HIV RNA copy numbers per cell, as well as cellular phenotype and activation level of single HIV-infected cells. A bulk HIV RNA load of 100 copies per million cells can mean that there are either 10 HIV RNA+ cells per million cells with 10 copies HIV RNA per cell, or 1 HIV RNA+ cell with 100 copies per cell. To discriminate between these possibilities, and to determine whether frequencies of HIV RNA+ cells or copy numbers per cell (or both) are changed upon ex vivo virus stimulation, a single-cell approach is necessary. Development of assays that would allow to characterize single HIV-infected cells, including the viral transcription level, is a top priority in the HIV cure field, and several groups recently reported the development of novel single-cell techniques for HIV RNA detection, based on either limiting dilution-PCR or in situ hybridization chemistries. These techniques are discussed below.

In fact, already in 2002 Fischer’s group reported the development of a limiting dilution-PCR-based assay to study the frequencies and expression levels of US and MS CA HIV RNA at the single-cell level [70], and this assay has been further developed throughout the 2000s [5, 71]. This large and unique body of work resulted in a number of insights into the persistence of viral transcription-active reservoirs in ART-treated individuals including the cellular origin and activation level of HIV RNA+ cells [5]. Notably, all these measurements have been done in the absence of any ex vivo stimulation. More recently, studies of HIV transcription under ART largely shifted to measuring its activation *in* and ex vivo, as well as estimating the replication-competent reservoir size, and in 2014, Cillo et al. reported the first inducible HIV transcription assay ([130], also see the review of Plantin et al. in this Special Issue [77]). This assay, based on limiting dilution-PCR, was primarily designed to measure the frequencies of HIV proviruses that could be reactivated to produce virions upon ex vivo stimulation, but for two donors, frequencies of cells reactivated to transcribe CA US RNA were also reported. These frequencies were 5–24 times higher than those of virion-producing cells, suggesting that most of HIV RNA+ cells cannot be reactivated for virion production due to post-transcriptional blocks or defective proviruses. Subsequently, Procopio et al. [78] reported the development of the *Tat/rev* Induced Limiting Dilution Assay, or TILDA, which is based on the modified version of our previously reported seminested RT-qPCR assay for MS RNA [122]. In brief, TILDA measures the frequencies of CD4+ cells that can be induced to produce MS RNA-*tat/rev*. Because no RNA extraction is performed and RT-PCR is done directly on cells, the assay is relatively fast, and despite its limiting dilution format it does not require large cell numbers. However, the downside of this approach is that because of inhibition of the RT and/or PCR step by cellular proteins, the maximal number of cells that can be assayed in one well is limited to 18,000, compromising the assay sensitivity. The limiting dilution format allows to alleviate this sensitivity issue by combining technical replicates, however this translates into wide confidence intervals for the final output measure, resulting in imprecise estimation of the cell frequencies that are close to the limit of detection. The absence of an RNA extraction step before RT-PCR also precludes the measurement of US RNA by TILDA, because no DNase treatment can be performed, which is necessary to remove the proviral DNA that is collinear with US RNA. The inducible Cell-Associated RNA Expression in Dilution assay (iCARED), developed by Massanella et al. [131], measures the frequencies of both US and MS RNA+ cells, in addition to virion-producing cells. Confirming the data of Cillo et al., this assay revealed that the frequency of cells producing US and MS RNA are 25- and 5-fold higher than the frequency of virion-producing cells. Interestingly, frequencies of cells that can be activated to produce US or MS RNA measured by TILDA or iCARED did not significantly correlate with virus outgrowth, suggesting that either the inducible HIV transcription assays, or the virus outgrowth assay (or both), are not very reliable as a measure of the replication-competent HIV reservoir. This is because any assay based on the inducible CA RNA+ cell measurement overestimates the reservoir as it still measures some defective proviruses, and the virus outgrowth assay underestimates the reservoir as only a fraction of intact proviruses could be induced at any time [11, 132].

One shortcoming of all the inducible HIV transcription assays described above is that they only report frequencies of HIV RNA+ cells but not HIV RNA copy numbers per cell, which would be useful to evaluate the effects of LRAs on the single-cell level, as it is unknown whether LRAs preferentially activate previously transcriptionally silent proviruses or boost already ongoing transcription. Wiegand et al. [133] recently developed cell-associated HIV RNA and DNA single-genome sequencing assay (CARD-SGS), which does not use quantitative PCR to directly measure CA RNA but rather estimates the frequencies of CA US RNA+ cells and RNA copy numbers per cell based on the assessment of a sequence match in the p6-PR-RT region of HIV (~ 1.3 kb). Briefly, SGS is performed on CA RNA isolated from aliquots diluted to contain 1–12 HIV RNA+ cells, and identical RNA sequences from the same aliquot are assigned to a single infected cell. By counting the number of different HIV variants in each aliquot, the fraction of infected cells that express HIV RNA is estimated, and the RNA copy numbers per cell are then estimated based on the numbers of identical sequences. Applying these analyses to PBMC isolated from four HIV-infected donors, Wiegand et al. determined that the average fraction of HIV-infected cells that express CA US RNA was 7%, which is close to earlier estimates [5, 71]. Furthermore, in the three ART-treated donors, a median of 29% of the HIV RNA-expressing cells had more than one HIV US RNA molecule detected, but none contained more than 10 molecules per cell. In the untreated participant, 56% of cells had more than one HIV RNA molecule, but only 2% had more than 10 HIV RNA molecules per cell. This per-cell US RNA content is lower than estimated earlier by Fischer et al. [71], possibly reflecting low donor numbers in both studies and/or different duration of ART. The advantage of this method is that CA RNA is sequenced, which allows determining the intactness/defectiveness, as well as clonal expansion, of the expressed proviruses. A limitation of the assay is that only 15% of HIV genome is sequenced and the rest of the genome might still contain sequence mismatches, therefore one cannot be sure whether the RNA molecules assigned to one cell do not in fact belong to different cells, which could affect the interpretation of the results. However, given the relatively high HIV nucleotide diversity in these donors, such bias might be small [134].

An extreme “limiting dilution” approach to characterize single HIV RNA+ cells was recently reported by Yucha et al. [135]. They adapted the digital PCR technique to develop an innovative microfluidic single-cell-in-droplet PCR assay to directly quantify the number of US RNA+ and MS RNA+ cells and the changes in these numbers upon reversion of latency. Individual cells are encapsulated into nanoliter-scale reaction droplets, lysed within droplets, and droplets are subjected to PCR amplification and sorted by fluorescence as in regular ddPCR. The results highlighted large inter-patient and inter-assay variation in the response to LRA. Although this assay is expected to be subject to the digital PCR limitations described above, the single-cell-in-droplet technique is promising and further research will show whether US and MS RNA measurements can be multiplexed within the encapsulated cell, and whether per-cell HIV RNA content can be determined in single-cell lysates isolated from positive droplets.

Apart from the PCR-based techniques, in situ hybridization (ISH)-based methods can be used to study HIV transcription (and translation) at the single-cell level. Already in the 1990s, several groups developed ISH-based assays to visualize HIV RNA+ cells [136, 137]. This method was shown to be biologically relevant as Derdeyn et al. demonstrated a near-perfect correlation between the frequencies of cells from HIV-infected donors that could be stimulated ex vivo to transcribe viral RNA, enumerated by ISH, and cells that could be stimulated to produce infectious virus, measured by a coculture assay [136]. At about the same time, Patterson and colleagues developed simultaneous ultrasensitive subpopulation staining/hybridization in situ assay (SUSHI), combining cell surface immunophenotyping with fluorescent ISH for US RNA [137, 138]. This assay has been used in several studies, which demonstrated the correlation of US RNA+ cell frequencies with ex vivo proliferative responses to HIV CA-p24 and confirmed our data on the clinical relevance of US RNA measurement for predicting the response to ART [139, 140]. More recently, next-generation ISH-based assays for HIV RNA detection have been reported, with HIV RNA+ cells either visualized by microscopy in tissue sections (RNAscope, Advanced Cell Diagnostics) or detected by flow in samples from peripheral blood (Human PrimeFlow RNA assay, Affymetrix/eBioscience) [40, 141–143]. These methods are reviewed in depth by Baxter et al. and Deleage et al. in this Special Issue [144, 145], therefore here we will not elaborate on the technology. A big advantage of the ISH-based methods is the possibility of phenotypic characterization of single HIV RNA+ cells, for example staining for various cell surface molecules, or even simultaneous detection of HIV proteins [40]. A disadvantage might be lower sensitivity compared to the PCR-based methods, as the presence of several target RNA molecules in a cell is required for that cell to be scored positive by ISH. Therefore, cells with the per-cell RNA content that is lower than the threshold might be missed. Indeed, both Grau-Esposito et al. and Deleage et al. observed 2–3 log_10_ lower HIV/SIV RNA+ cell numbers measured by their assays than the bulk HIV/SIV RNA levels quantified by qPCR [141, 142], suggesting that only cells with high per-cell viral RNA content are detected. Another disadvantage could be the high background signal level in the HIV RNA channel that can compromise the specificity when measuring rare HIV RNA+ cells in ART-treated individuals [40, 142]. Of note, inducible HIV RNA+ cell frequencies measured by both PrimeFlow-based assays did not correlate with quantitative virus outgrowth values [40, 142].

## Defective versus intact: Is it worth measuring at all?

More than 90% of proviruses in ART-treated individuals have been shown to be defective at the sequence level for replication-competent virus production due to large internal deletions, hypermutation, premature stop codons, defects in major splice donor site or packaging signal, etc. [132, 146–148]. Moreover, even the small minority of “intact” proviruses might overestimate the size of the replication-competent reservoir as not all defects can be readily determined by sequence analysis (but on the other hand, HIV may overcome some of the defects that are obvious by sequence analysis by using alternative ways to express its genes [149, 150]). For the correct interpretation of CA RNA measurements, it is important to understand whether the fraction of HIV RNA that is defective is smaller or larger than the defective provirus fraction. In other words, it is still unclear to what extent the ability to transcribe HIV RNA is independent of the intactness of the provirus. On one hand, HIV US RNA transcription requires at least a functional LTR, and other *cis*-acting signals are necessary for production of spliced RNA forms (reviewed in [59]). On the other hand, cells that produce intact CA RNA, or are capable of doing so upon activation, might be preferentially recognized and eradicated by the host immune system as they can present viral antigens. It has been demonstrated that defective proviruses can be transcribed and translated, which can lead to the CTL recognition [133, 151–153]. Two recent studies from Palmer’s group reported larger fractions of defective CA RNA compared to defective DNA in ART-treated individuals before and after LRA treatment [154, 155], indeed suggesting the selective elimination of intact CA RNA-producing cells by host immunity. However, only a small HIV region (V1–V3 region of *env*) was sequenced in these studies, and therefore only hypermutations and premature stop codons could be assessed, leaving the possibility that the relative ratio between defective CA RNA and defective HIV DNA fractions is different on the full-genome level.

**Fig. 1:**
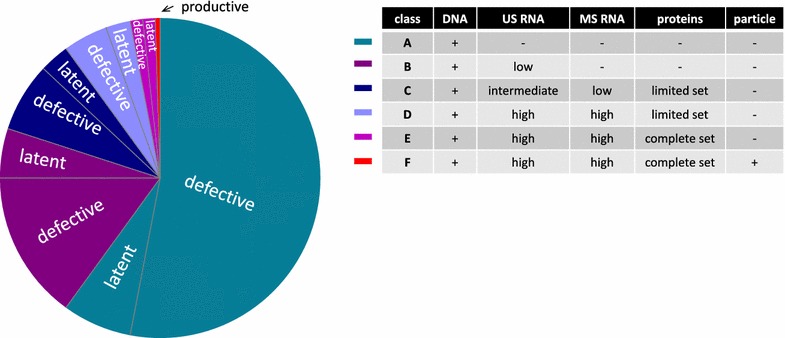
Estimation of the relative contribution of putative cell classes defined by reversible inhibition (latent infection) or irreversible blocks (defective infection) of different stages of HIV expression to the total pool of HIV-infected cells in ART-treated individuals. (A) HIV-infected cells that do not transcribe any CA RNA species due to the lack of transcription initiation factors, chromatin organization, epigenetic modifications, etc. (latent infection), or sequence defects in the LTR promoter, Tat-TAR defects, etc. (defective infection). (B) Cells that contain abortive transcripts and low levels of US RNA in the nucleus (which may be incomplete) but no MS RNA and no HIV proteins, due to either lack of factors necessary for transcription elongation or splicing (latent infection), or deletions and splice site mutations (defective infection). (C) Cells that contain low levels of MS RNA, as well as intermediate levels of US RNA, some of which can be transported to the cytoplasm, and a limited set of HIV proteins, due to either low levels of splicing or nuclear export factors (latent infection), or deletions, hypermutation, and mutations in splicing enhancer sequences or in Rev response element (defective infection). (D) Cells that contain high levels of both US and MS RNA but express a limited set of HIV proteins, due to either inhibition of HIV translation by microRNAs or other host factors (latent infection), or deletions, frameshift mutations, and premature stop codons (defective infection). (E) Cells that contain high levels of both US and MS RNA and express the complete set of correct viral proteins but do not produce infectious particles due to either inhibition of particle assembly/maturation by host defense (latent infection), or mutations in the packaging signal (defective infection). (F) Cells that are productively infected. Note that the relative contributions of these cell classes to the total pool of HIV-infected cells, as well as the relative contributions of latent versus defective infection to each class, are rough estimates that are expected to differ substantially from patient to patient and might change over time on therapy, and other cell classes might be present

The transcription and translation capability of defective proviruses suggests, in addition to the “continuum of latency” discussed above, an existence of the “continuum of defectiveness”, with some defective proviruses being transcriptionally silent, some transcription-competent but defective for viral protein production, some capable of producing certain viral proteins but not others, some capable of producing all viral proteins but having a defect in the packaging signal, some producing non-infectious viral particles, etc. (Fig. 1). The presence of large deletions in CA RNA also means that any assay based on the measurement of only one genomic region will seriously underestimate the CA RNA level. A possible exception to this are assays based on exon–exon boundary-spanning amplicons that are used for spliced RNA measurements, but even in this case, one must be cautious as HIV can bypass defects in canonical splice sites, including the major splice donor, by using novel alternative spliced sites [149, 152]. Interestingly, Rassler et al. [156] reported prolonged persistence of an HIV variant with the mutated major splice donor site in plasma of a patient on suppressive ART, suggesting that HIV might have found alternative means to express spliced RNAs needed for virion production.

A very important issue relevant to current discussions on residual HIV pathogenesis in ART-treated individuals, which is only beginning to receive attention, is whether the defective proviruses can be pathogenic. Imamichi et al. [157] introduced the term “zombie proviruses”, implying that some defective proviruses can still cause harm despite being “dead”. Indeed, a number of studies demonstrated a correlation between the CA RNA levels and markers of immune activation and dysfunction under ART [66, 72, 158, 159], although other investigators challenged this view [160]. Although correlation does not imply causation and increased CA RNA levels might be not only the cause but also the consequence of e.g. increased immune activation, these results are at least suggestive of some biological function of defective proviruses that are expressed. Even if, due to deletions and/or frameshift mutations, no correct HIV proteins can be translated, a cell that expresses a foreign antigen can still be potentially recognized by the host immune system. In theory, persistence and multiplication of such “zombie” proviruses by clonal expansion increases the probability of causing elevated immune activation. In this sense, the “block-and-lock” strategy to permanently suppress CA RNA transcription [161, 162] might be useful even as an addition to conventional ART. Moreover, Li et al. [163] recently reported that acitretin, a retinoic acid derivative, both increases HIV transcription and induces preferential apoptosis of HIV-infected cells by the RIG-I pathway that involves recognition of HIV RNA. In theory, this strategy should eliminate cells harboring not only intact, but defective proviruses as well, provided the latter are expressed or have an ability to be expressed upon activation. Further research will show whether these strategies will result in decreased residual immune activation and inflammation in ART-treated individuals.

## Conclusions

In summary, CA HIV RNA as a biomarker of HIV persistence and latency reversion has received much interest in recent years, but at the same time it has become clear that the size of the HIV transcription-competent reservoir overestimates the size of the replication-competent reservoir as a substantial (but yet unknown) fraction of CA RNA-transcribing proviruses is likely defective for infectious virion production. Does this mean that CA RNA is not informative and that we should stop measuring it? We would argue against such a view, as (1) CA RNA has been shown to be a biomarker that is significantly more sensitive for monitoring ART and predicting virological failure than plasma viremia, at least when the latter is measured by commercial assays [3, 4], (2) even defective expressed proviruses might contribute to residual HIV pathogenesis [151, 152], (3) the “baseline” level of CA RNA correlates with the level upon LRA treatment [6], suggesting that CA RNA level might serve as a predictor of the efficiency of latency reversal and that future treatments could be tailored to individual patients [164], (4) transcription-competent proviruses under ART contribute to the virus rebound after therapy interruption, as reported by different groups [153–155], (5) in addition to the LRA studies, CA RNA can be used as a surrogate marker of the efficacy of antiviral gene therapy strategies, in particular CRISPR/Cas9 gene editing [165, 166], and (6) probably most importantly, CA RNA levels measured by a simple bulk PCR-based assay (without ex vivo stimulation) at ART interruption are predictive of the duration of post-treatment control, as independently reported by Li et al. and our group [167, 168]. By mathematical modeling, the duration of post-treatment control (ART-free HIV remission) has been shown to be a direct reflection of the size of replication-competent viral reservoir, and probably its best measure that currently exists [169–171]. Therefore, any biomarker that reliably correlates with the time to viral rebound should be used as a surrogate marker of the replication-competent reservoir, and as ART interruption is currently the only way to determine whether a patient is cured or whether a curative treatment has been effective, biomarkers that could predict the duration of ART-free remission are urgently needed. Both CA RNA and total HIV DNA have been shown by different groups to predict the time to viral rebound [167, 168, 172] and further research should indicate whether these associations are sufficiently robust for these markers to support the clinical decision-making on the ART interruption during HIV cure-related clinical trials [94], and to allow initial assessment of novel curative interventions without the need for ART interruption. Without doubt, identifying the predictors of ART-free remission, and therefore the correlates of the replication-competent HIV reservoir size, will greatly facilitate progress in the HIV cure field.

## Abbreviations

CA: Cell-associated
HIV: Human immunodeficiency virus
ART: Antiretroviral therapy
LRA: Latency-reversing agent
PBMC: Peripheral blood mononuclear cell
CROI: Conference on retroviruses and opportunistic infections
CCL19: Chemokine (C-C motif) ligand 19
PSF: Polypyrimidine tract binding protein associated splicing factor
CTL: Cytotoxic T lymphocyte
US: Unspliced
MS: Multiply spliced
PMA: Phorbol 12-myristate 13-acetate
RT-qPCR: Reverse transcription-quantitative polymerase chain reaction
NF-κB: Nuclear factor kappa-light-chain-enhancer of activated B cells
GALT: Gut-associated lymphatic tissue
SIV: Simian immunodeficiency virus
SHIV: Simian-human immunodeficiency virus
CCR7: C-C chemokine receptor type 7
PD-1: Programmed cell death protein 1
CXCR5: C-X-C chemokine receptor type 5
T_FH_: Follicular T helper
ddPCR: Droplet digital PCR
TILDA: *Tat/rev* induced limiting dilution assay
iCARED: Inducible cell-associated RNA expression in dilution assay
CARD-SGS: Cell-associated HIV RNA and DNA single-genome sequencing assay
ISH: In situ hybridization
SUSHI: Simultaneous ultrasensitive subpopulation staining/hybridization in situ assay
LTR: Long terminal repeat
RIG-I: Retinoic acid-inducible gene I
